# Short read Illumina data for the *de novo *assembly of a non-model snail species transcriptome (*Radix balthica*, Basommatophora, Pulmonata), and a comparison of assembler performance

**DOI:** 10.1186/1471-2164-12-317

**Published:** 2011-06-16

**Authors:** Barbara Feldmeyer, Christopher W Wheat, Nicolas Krezdorn, Björn Rotter, Markus Pfenninger

**Affiliations:** 1 Biodiversity and Climate Research Center (BiK-F), Molecular Ecology Group, Biocampus Siesmayerstraße, Goethe-Universität, 60323 Frankfurt am Main, Germany; 2 Department of Biological and Environmental Sciences, Viikinakaari 1, FI-00014 University of Helsinki, Finland; 3 GenXPro, Altenhöferallee 3, Frankfurt am Main, Germany

**Keywords:** next generation sequencing, short read assembly, Mollusca

## Abstract

**Background:**

Until recently, read lengths on the Solexa/Illumina system were too short to reliably assemble transcriptomes without a reference sequence, especially for non-model organisms. However, with read lengths up to 100 nucleotides available in the current version, an assembly without reference genome should be possible. For this study we created an EST data set for the common pond snail *Radix balthica *by Illumina sequencing of a normalized transcriptome. Performance of three different short read assemblers was compared with respect to: the number of contigs, their length, depth of coverage, their quality in various BLAST searches and the alignment to mitochondrial genes.

**Results:**

A single sequencing run of a normalized RNA pool resulted in 16,923,850 paired end reads with median read length of 61 bases. The assemblies generated by VELVET, OASES, and SeqMan NGEN differed in the total number of contigs, contig length, the number and quality of gene hits obtained by BLAST searches against various databases, and contig performance in the mt genome comparison. While VELVET produced the highest overall number of contigs, a large fraction of these were of small size (< 200bp), and gave redundant hits in BLAST searches and the mt genome alignment. The best overall contig performance resulted from the NGEN assembly. It produced the second largest number of contigs, which on average were comparable to the OASES contigs but gave the highest number of gene hits in two out of four BLAST searches against different reference databases. A subsequent meta-assembly of the four contig sets resulted in larger contigs, less redundancy and a higher number of BLAST hits.

**Conclusion:**

Our results document the first *de novo *transcriptome assembly of a non-model species using Illumina sequencing data. We show that *de novo *transcriptome assembly using this approach yields results useful for downstream applications, in particular if a meta-assembly of contig sets is used to increase contig quality. These results highlight the ongoing need for improvements in assembly methodology.

## Background

Reliable annotation of cDNA sequences is of great concern to research groups working with non-model organisms, in particular in the emerging field of ecological genomics [1-3]. Thus, many groups still make use of Sanger sequencing technologies in order to obtain long reads which can be reliably annotated [4-8]. However, recently Next Generation Sequencing (NGS) techniques have become a valuable tool for transcriptome analysis. Nearly all *de novo *transcriptome sequencing projects to date using NGS have employed the 454/Roche system which provides considerably longer read lengths compared to other NGS methods at the time [9-11]. Solexa/Illumina transcriptome studies on the other hand have been limited to species with an available genome to use as a reference sequence for read assembly [12,13]. Recently Gibbons et al. [14] created non-normalized *Aedes aegypti *and *A. gambiae *EST-libraries by Solexa/Illumina sequencing. Although their estimated coverage of the transcriptome was low (i.e. < 30%), their assembled contigs were very accurate and informative [14]. They concluded that their obtained sequence length (~75bp), together with the vast amount of reads, made this method suitable for contig assembly from transcriptome data in non-model organisms. Similarly, the first *de novo *transcriptome assembly paper based on the first generation of 454 sequencing technology achieved relatively good assembly performance, with a large number of positively identified contigs in BLAST comparisons versus several insect databases, and reads that were on average only 110bp long [11]. Together these two studies suggest that as the Solexa/Illumina technology gets closer to 100bp in length, this technology could generate suitable data for a high quality transcriptome assembly with nearly 20 times the coverage for roughly 1/10 the current cost of a given 454/Roche run. Here we demonstrate for the first time the use of Solexa/Illumina data for *de novo *assembly on a non-model species, but also that short read assembly algorithms first need to improve to make this the platform of choice.

NGS platforms present an important tradeoff between the number of fragments sequenced per run and the read length per fragment. A full 454/Roche-system run produces around one million reads that have an average read length of about 400bp. Systems like Solexa/Illumina and Solid/ABI on the other hand produce close to 20 million reads per lane, with sequences about 50-100bp long. Importantly, a single lane of Solexa/Illumina sequencing is substantially cheaper than a full 454 run. While sequence reads are a valuable resource by itself, short reads are most useful for downstream applications (e.g. sequence annotation) if they are assembled accurately and efficiently [15]. Assembly by itself is a commonly overlooked yet critical step, especially when working with NGS data in species without a reference genome [3]. While much of the ecological genetics research community is focused upon read lengths, assembly annotation, and SNP identification, optimization of data assembly needs more attention. Here we address these needs by evaluating the performance of short read assemblers in *de novo *transcriptome assembly of Illumina sequence data without a reference genome.

Genomic tools are needed in order to study local adaptation and the adaptive potential of the common pond snail *Radix balthica*. *R. balthica *is distributed across northern Europe and is primarily found in slow flowing water bodies and lakes [16,17]. Based on its mixed mating system *R. balthica *is an interesting study organism to investigate sex allocation, mating strategies, sperm allocation and sexual conflict. Recently it has been shown that the distribution of *R. balthica *is shifting northwards, following temperature shifts caused by global warming [18]. While the natural history and distribution of this species render it a good study species, it currently lacks sufficient genomic tools to thoroughly investigate these issues.

Here we use the Illumina/Solexa sequencing platform to sequence a normalized cDNA pool from the common pond snail *R. balthica*. Since this platform has not previously been used for *de novo *transcriptome assembly on a normalized cDNA pool, we compare and evaluate the usefulness of three different assemblers. Our findings indicate significant differences among assemblers and highlight the potential of using Illumina sequencing for non-model species.

## Results

### Illumina sequencing

The normalized RNA pool yielded 16,923,850 reads with a median of 61 nucleotides in length and a maximum read length of 101bp. The raw sequence data are available at NCBI Short Read Archive (SRP005151). The 13 mtDNA genome gene sequences of *R. balthica *were used as reference to calculate transcript coverage, which was found to range between 5x for the ND4L gene up to 989x for COIII, with a mean coverage of 137x. One means to asses normalization success, is to determine whether the average number of ESTs per ribosomal gene contigs does not exceed 5x the average number of ESTs for all other contigs [3]. Using the NGEN assembly, there was an average of 487 ESTs per ribosomal gene contig vs. 541 for the non-ribosomal genes, indicating that normalization was successful.

### Indirect contig quality assessment

#### Velvet

**Figure 1:**
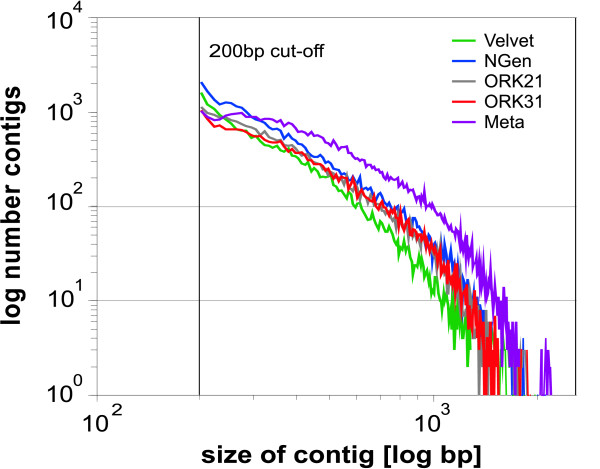
**Distribution of contig number and length (contigs > 200bp)**. Total number of contigs obtained by each assembler: VELVET = 220.154; NGEN = 57,986; OASES*k*mer-21 = 52,477; OASES*k*mer-31 = 41,590; Meta-assembly = 54,450.

**Figure 2:**
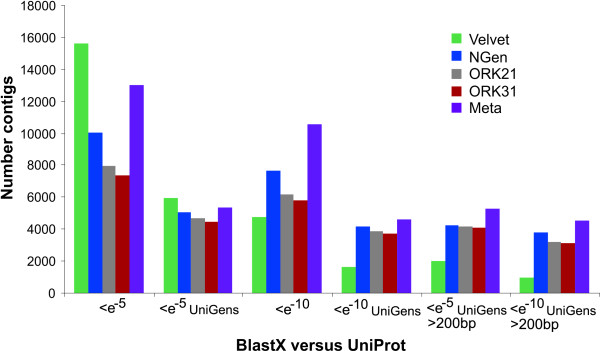
**Contig BLASTX hits against the UniProt database**. Overview BLAST results of contigs produced by three different assemblers. BLASTX cutoff values were set to either < e^-5 ^or < e^-10^. Results are shown for the total number of hits, the number of UniGen hits, and for contigs larger than 200bp. (ORK21/31: OASES*k*mer-21/31).

**Figure 3:**
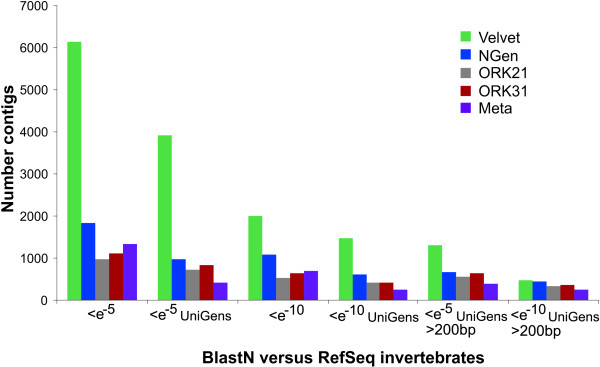
**Contig BLASTN hits against the RefSeq database**. Overview BLAST results of contigs produced by three different assemblers. BLASTN cutoff values were set to either < e^-5 ^or < e^-10^. Results are shown for the total number of hits, the number of UniGen hits, and for contigs larger than 200bp. (ORK21/31: OASES*k*mer-21/31).

**Figure 4:**
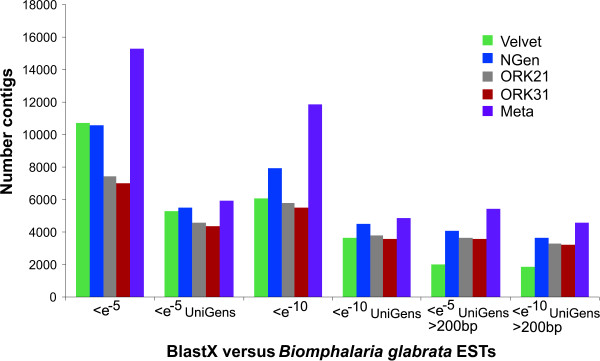
**Contig BLASTX hits against the *Biomphalaria glabrata *EST database**. Overview BLAST results of contigs produced by three different assemblers. BLASTX cutoff values were set to either < e^-5 ^or < e^-10^. Results are shown for the total number of hits, the number of UniGen hits, and for contigs larger than 200bp. (ORK21/31: OASES*k*mer-21/31).

The VELVET assembly resulted in a total of 220,153 contigs ranging in size between 61bp to 2257bp, with a mean of 120bp in length (Table 1, Figure 1). Only 9% of these contigs were longer than 200bp. A BLASTX against the UniProt database with a cutoff value of <*e*^-5 ^yielded 115,649 putative gene homologies, which resulted in 5956 unique gene hits (UniGens) after removing redundant hits (Figure 2). A cutoff value of <*e*^-10 ^decreased the number of hits by over one third to 4736, leading to 1671 UniGens, of which 962 are above 200bp. A BLASTN against the RefSeq Invertebrate database with a cutoff value <*e*^-5 ^yielded 6127 putative gene similarities, which after removing redundant hits resulted in 3907 UniGens (Figure 3). Most of these hits matched to the sea urchin *Strongylocentrotus purpuratus *(10%), followed by the sea anemone *Nematostella vectensis *(7%) and *Hydra magnipapillata *(7%). In total, gene homologies were identified across 32 different species, of which 23 were insect species. Applying a cutoff value of <*e*^-10 ^the BLASTN against the RefSeq Invertebrates database resulted in 2010 hits of which 1465 are UniGen hits and 475 are larger than 200bp. A BLASTX against *Biomphalaria glabrata *ESTs yielded 10,750 hits with <*e*^-5 ^and 5269 UniGens (Figure 4). Decreasing the cutoff value to <*e*^-10 ^rendered 6055 hits with 3621 UniGens and 1866 longer than 200bp.

**Table 1:** Summary of contig length properties for the total number of contigs as well as contigs larger than 200bp, resulting from different assemblers and meta-assembly, respectively

	all contigs					> 200bp contigs				
	
	Velvet	NGen	ORK21	ORK31	Meta	Velvet	NGen	ORK21	ORK31	Meta
Min	61	31	55	52	61	200	200	200	200	200
Max	2257	2435	2070	2551	2667	2257	2435	2070	2551	2667
Mean	120	303	267	309	432	398	428	442	443	536
Median	93	217	179	213	341	326	338	359	359	442
Total	220,153	57,986	52,477	41,590	54,450	20,346	31,898	23,816	22,089	39,941

(ORK21/31: OASES*k*-mer = 21/31)

#### Oases

The OASES assembly with *k*-mer = 21 resulted in a total of 52,477 contigs with mean length 267bp; *k*-mer = 31 yielded 41,590 contigs with mean length 309bp (Table 1, Figure 1). Both *k*-mer lengths resulted in more than 20,000 contigs larger than 200bp which corresponded to 45% and 53% of the total number of contigs produced. As the BLAST results of the OASES assemblies with different *k*-mer sizes lead to comparable results, we only discuss the outcome of *k*-mer size 31 in detail (Figures 2, 3 and 4 for further information). Subjecting the contigs to a BLASTX against the UniProt database with a cutoff value of <*e*^-5 ^yielded 7347 putative gene homologies, which resulted in 4426 UniGens after removing redundant hits (Figure 2). A BLASTN against the RefSeq Invertebrate Database with a cutoff value <*e*^-5 ^yielded 1,112 putative gene similarities, which after removing redundant hits resulted in 824 UniGens (Figure 3). Overall homologies to 38 different species were detected, of which 23 belonged to insect species. In contrast to the VELVET results, three mitochondrial homologies belonging to snail species were detected. Most of the hits matched to the sea urchin *Strongylocentrotus purpuratus *(19%), followed by *Hydra magnipapillata *(12%), and the sea anemone *Nematostella vectensis *(8%). Using a cutoff value of <*e*^-10 ^decreased the number of RefSeq hits to 5821 leading to 3691 UniGens, of which 3126 are above 200bp. Applying a cutoff value of <*e*^-10 ^resulted in 642 hits of which 414 are UniGen hits and 349 are larger than 200bp. A BLASTX against *Biomphalaria glabrata *ESTs yielded 7023 hits with <*e*^-5 ^and 4331 UniGens (Figure 4). Decreasing the cutoff value to <*e*^-10 ^rendered 5512 hits with 3574 UniGens and 3182 lager than 200bp.

#### NGen

**Figure 5:**
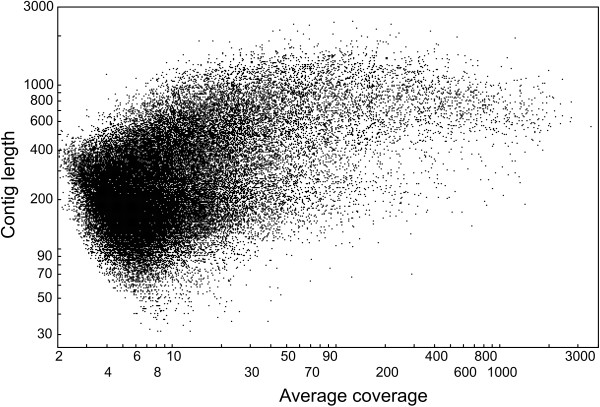
**Distribution of contig length versus average coverage, shown for the NGEN assembled contig set**.

The NGEN assembly resulted in a total of 57,986 contigs with mean length 303bp (Table 1, Figure 1). Fifty-five percent of the total contigs were found to be larger than 200bp. Figure 5 depicts the relationship of average coverage versus contig size. Contig length increased with coverage depth and reached an asymptote approximately at an average coverage of about 30. A BLASTX against the UniProt database with a cutoff value of <*e*^-5 ^yielded 10,072 putative gene homologies, which resulted in 5059 UniGens after removing redundant hits (Figure 2). Using a cutoff value of <*e*^-10 ^resulted in 7630 hits and 4166 UniGens, of which 3810 were larger than 200bp. A BLASTN against the RefSeq Invertebrate database with a cutoff value <*e*^-5 ^yielded 1835 putative gene similarities, which after removing redundant hits resulted in 974 UniGens (Figure 3). Most of the hits matched to *Hydra magnipapillata *(16%), followed by the sea urchin *Strongylocentrotus purpuratus *(12%), and the tick *Ixodes scapularis *(9%). Overall homologies to 44 different species were detected, of which 21 belonged to insect species, but also 12 mitochondrial homologies were found belonging to 11 snail species. Applying a cutoff value of <*e*^-10 ^decreased the number of RefSeq hits to 1091 of which 606 are UniGen hits and 441 were larger than 200bp. A BLASTX against *Biomphalaria glabrata *ESTs yielded 10,569 hits with <*e*^-5 ^and 5490 UniGens (Figure 4). Decreasing the cutoff value to <*e*^-10 ^rendered 7913 hits with 4513 UniGens and 3671 lager than 200bp.

#### Meta-assembly

The NGEN meta-assembly resulted in a total of 54,450 contigs with mean length 432 bp (Table 1, Figure 1). Seventy-three percent of the total contigs were found to be larger than 200bp. A BLASTX against the UniProt database with a cutoff value of <*e*^-5 ^yielded 13,002 putative gene homologies, which resulted in 5385 UniGens after removing redundant hits (Figure 2). Using a cutoff value of <*e*^-10 ^resulted in 10,553 hits and 4,590 UniGens, of which 4547 were larger than 200bp. A BLASTN against the RefSeq Invertebrate database with a cutoff value <*e*^-5 ^yielded 1334 putative gene similarities, which after removing redundant hits resulted in 415 UniGens (Figure 3). Most hits were to the sea urchin *Strongylocentrotus purpuratus *(18%), followed by the honey bee *Apis mellifera *(13%), and the jewel wasp *Nasonia vitripennis *(9%). Applying a cutoff value of <*e*^-10 ^decreased the number of RefSeq hits to 702 of which 257 were UniGen hits and 247 larger than 200bp. A BLASTX against *Biomphalaria glabrata *ESTs yielded 15,266 hits with <*e*^-5 ^and 5949 UniGens (Figure 4). Decreasing the cutoff value to <*e*^-10 ^rendered 11,834 hits with 4890 UniGens and 4598 lager than 200bp.

#### Assessing UniProt hits

**Figure 6:**
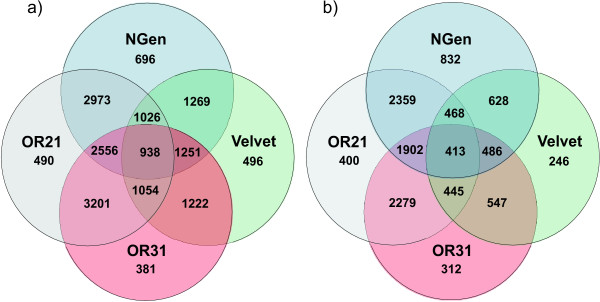
**Number of unique and identical UniProt gene hits of the four different contig sets**. BLASTX applied cutoff values a) < e^-5 ^and b) < e^-10 ^with contigs > 200bp.

In the absence of genomic sequence for *R. balthica*, contigs from the different assemblies were compared among each other. Only contigs having UniProt BLASTX gene hits were used. Pairwise comparisons of single gene hits (contigs > 200bp) revealed higher a similarity between the NGEN and OASES contig sets (both *k*-mer sizes), than between VELVET and any of the latter (Figure 6). This was true for both cutoff values. For example, NGEN shared 2973 hits with OASES*k*-mer = 21 with a cutoff value of <*e*^-5^, but only 1269 with VELVET. With a cutoff value of <*e*^-10 ^NGEN shared 2359 hits with OASES*k*-mer = 21, and 628 with VELVET.

### Direct contig quality assessment via mitochondrial genes

Assemblies with shorter contigs had more hits against the mtDNA genes, with VELVET resulting in the highest number of hits (560), but the shortest total and aligned contig lengths (107 and 45 bp, respectively; Table 2). The meta-assembly had the fewest number of hits and the longest contigs. This relationship is also reflected in the number of contigs with a > 80% identity in their alignment against the mtDNA genes, as shorter contigs gave more hits. Yet, on the average, the amount each contig assembly covered these mtDNA genes was rather similar at around 50 to 55% coverage (Table 2; Additional file 1).

**Table 2:** Performance of contigs BLASTN against the 13 *R. balthica *mitochondrial genes with a cutoff value of < e^-5^

	Velvet	NGen	ORK21	ORK31	Meta
Total number hits	560	209	85	99	82
Average total contig length	107	248	259	255	475
Average aligned contig length	45	90	83	79	216
# of contigs with > 80% identity	214	21	15	19	21
# of contigs with 100% identity	21	0	0	0	0
Summed aligned coverage	5696	5610	5332	5527	6143
Average % coverage per gene	0.55	0.54	0.51	0.53	0.58

Summed aligned coverage indicates the sum of the aligned region of contigs across the 13 mitochondrial genes. (ORK21/31: OASES*k*-mer = 21 & 31).

**Figure 7:**
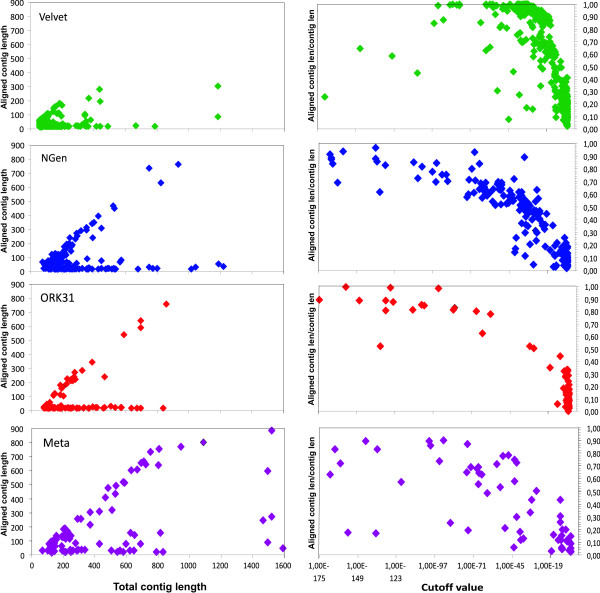
**Contig quality assessment by BLASTN against *Radix *mt genes**. Relationship aligned contig length versus total contig length (left panel), as well as proportion of aligned contig length versus total contig length and cutoff value < e^-5 ^(right panel). As the pattern of OASES*k*mer-21 and 31 contigs are similar only OASES*k*mer-31 is depicted here.

Contig assembly was also assessed by looking at the average aligned contig length vs. the total contig length (Figure 7, left panels). VELVET contigs are all very short and show little relationship between increasing contig length and increased alignment length, while this relationship is very clear for the other assemblies. Directly related to this is the ratio of the aligned region to the full length of a given contig, plotted against decreasing cutoff value alignment score (Figure 7, right panel). All but the meta-assembly show an increase in this ratio as alignment score increases (cutoff value decreasing), with the meta-assembly having several outlier contigs with good alignment scores but low ratio. These outliers appear to be chimeric contigs, consisting of two previous contigs incorrectly joined together.

### Functional annotation of ESTs

Using BLAST2GO (version 2.4.4), we were able to assign gene ontology classes to 2270 of the 2405 BlastX hits identical between the NGEN and OASES*k*-mer = 31 assemblies. There were a total of 30,464 gene ontology (GO) terms on all GO-levels associated with the 2270 identified genes. Of these, assignments to level two GO-terms of the highest order category Biological Processes made up the majority (5652) followed by Cellular Components (4231) and Molecular Function (3124) (Additional file 2).

### Repeat motifs

As shown previously, transcriptome sequences are useful to identify repeat motifs (i.e. microsatellites), which due to their high levels of variation are widely used for studies such as genome mapping, parentage analysis, analysis of gene flow and genetic structure [e.g. 1, 14, 19-21]. Using the program Sputnik [22], we identified 552 di-, tri-, tetra-, and penta-nucleotide repeats with a minimum repeat length of six, respectively (Additional file 3).

## Discussion

Accurate sequence reads and their reliable assembly is crucial for all downstream applications of NGS projects [15]. Without a reference genome, estimating the number of genes sequenced, their % coverage, and whether they have been assembled correctly is challenging [3,23]. As of the use of NGS continues to increase for non-model organisms, the need for assembly algorithms that perform well in *de novo *assembly concomitantly increases, especially for the assembly of the short read sequence data for the Solexa/Illumina platform [3].

The performance of the three short read assemblers (VELVET, NGEN and OASES) investigated differed greatly. While VELVET resulted in the highest number of total contigs, only nine percent of these were larger than 200bp. In contrast, over 50% of the NGEN and OASES assembled contigs were larger than 200bp. As mapping accuracy increases with increasing contig size [14], we reason that the latter contig sets should resemble higher overall quality. This assumption was strengthened by the results of the BLAST searches. Meta-assembly of the four contig sets resulted in longer contigs, which also result in a higher number of BLAST hits in most searches.

### Indirect contig quality assessment

Although the VELVET assembly had the largest number of contigs and the greatest number of hits against various databases, these are due to the poor assembly of contigs. Importantly, our ability to gain this insight is dependent upon the reference database used for BLAST searches and thus requires careful attention. In the BLAST comparisons against the UniProt database, the number of UniGen hits for the VELVET contigs is substantially smaller compared to the other assemblies with a cutoff value of <*e*^-10 ^and also contigs > 200bp (Figure 2). The discrepancy between total and UniGen hits derives most probably from the incomplete assembly of contigs by the VELVET assembler, resulting in many independent contigs each hitting similar genes, while these are joined together by the other programs and thus constitute single hits to given genes for the NGEN and OASES assemblies. Additionally, the long contig assemblies by the other programs generate more high quality BLAST hits than those found for VELVET (Figure 2). However, BLAST results against RefSeq indicate a much greater number of UniGen hits by the VELVET assembly than the other two methods (Figure 3). This result arises due to the highly redundant nature of the RefSeq database, as it contains unique sets of genes for numerous species. The RefSeq database should therefore be used with caution since the number of unique types of genes should not differ significantly from those identified using the UniProt database.

In the BLAST comparison against a database consisting of a single closely related species, *B. glabrata*, the NGEN assembly resulted in the highest number of UniGens, and VELVET showing especially poor performance when considering assembled contigs > 200bp in length.

Combining all of these assemblies into the meta-assembly resulted in contigs that outperformed the other assemblies in the BLASTX searches against the UniProt and *B. glabrata *databases in all but one category.

An additional means of assessing contig assembly performance is to compare the actual hits identified by the different assemblies. Similar hits indicate similar contig sequence and accuracy. Comparisons were made between assemblies for the BLASTX search versus the UniProt database (cutoff value <*e*^-10^, contig length > 200bp), which showed that the proportion of contigs leading to identical gene hits was highest between the NGEN and OASES contig sets. This again strengthened our interpretation that the quality of the NGEN and OASES contigs exceeded those of VELVET.

### Direct contig quality assessment

The different contig assemblies were directly assessed by comparing their performance among the 13 mitochondrial genes of *R. balthica *[24]. In general, VELVET contigs had the highest number of hits against these genes due to these contigs being much shorter. The other assemblies had longer and fewer contigs, which had higher average alignment length, with the meta-assembly showing the best performance (i.e. fewest number of contigs with highest average aligned contig length (Table 2). Longer contigs had a lower identity match to the mtDNA genes, which likely arises due to genetic differences in the samples used for this and the published mtDNA genome for *R. balthica *and potentially sequencing errors (which have a higher probability to occur in long contigs compared to short ones). We identified some contigs whose middle region did not resemble the reference sequence and we identified these as assembly errors. In addition, most contigs of the NGEN and OASES assembly had a 20-30bp extension attached at the beginning of the contig that does not match the mtDNA genome. For the NGEN assembly, this extension was identified as the Illumina sequencing adaptor not removed during filtering due to low identity matching. For the OASES contigs we currently lack an explanation for the attachment origin. As the extensions seem to be an almost systematic error, cutting the first 30bp of each contig sequence is one means to solve this problem (although some good quality sequence may be lost).

Despite these differences, coverage of the mtDNA genes was quite similar among the contig assemblies, averaging around 50 - 55% (Table 2). Pooling all the contigs from the assemblies covered 79% of the mt genes. Thus, even though contigs of the three assemblers overlap to a large extent, each contig set covers some parts which are missed by the others, with at least 24% of the available bp information is not used by any of the three assemblers. We identified 27 clusters with 2 to 25 overlapping, and to a large extent identical VELVET contigs. In contrast, among the NGEN contigs not more than two contigs with more than 30bp overlap were found. As the two main reasons for insufficient assembly visual inspection of the mt genome alignments revealed insufficient read overlap and missed assemblies, even though identical and sufficient overlap was present. This might be traced back to the use of RNA from several pooled individuals, which leads to a larger number of SNP variants, and thus might hamper assembly [11]. In our study we identified 6.3 SNPs per thousand base pairs (n = 52), similar to the 6.7 identified SNPs in the Vera et al. [11] study. The estimated number of sequencing errors is almost identical (n = 51), and results in a sequencing error rate of 0.6%. Obviously SNP variation and sequencing errors affect the VELVET assembly, but do not appear to influence the other two assemblers. The meta-assembly combined the short VELVET SNP containing contigs into one, thus largely eliminating redundancy (Additional file 4). However, although the meta-assembly decreased the number of contigs from 560 to 82, this only resulted in a modest improvement in net coverage compared to VELVET (58% vs. 55% respectively).

Two other important observations merit discussion. First, contigs giving a hit against the mt genes can be split in two groups. One group of the contigs shows a clear relation between alignment length of the contig to total contig length. The other group consists of contigs that passed the cutoff value <*e*^-5^, but only have a very short alignment length to the reference sequence and are therefore due effectively due to random, non-homologous matches (Figure 7). Second, while a clear relationship between cutoff value and alignment length is visible for the NGEN and OASES contigs, both the VELVET and meta-assembly contigs have clear outliers that may be assembly errors. These are contigs near the cutoff value with low alignment length, and with very high stringency cutoff values (e.g. <*e*^-65^).

### Comparison to other studies

The number of UniGen matches against the UnipProt database found in other transcriptome studies of non-model organisms based on the 454/Roche platform is roughly similar to the 5380 meta-assembly matches detected in this study, at a cutoff value of <*e*^-5 ^(e.g. [11]*Melitaea cinxia*: 6122 at <*e*^-5 ^). However, given our increased sequencing effort compared to previous studies (total quality data produced: 976 Mbp vs. 66 Mbp, i.e. 14 times higher compared to the *M. cinxia *study [11]), we expected to identify more genes. Previous observations of low blast results in mollusk species can be traced back to three main factors [25,26]. First, the low amount of hits can be explained by the lack of EST datasets from mollusk species in Genbank [25,26], and the general paucity of mollusk genetic data compared to insects and fish. Second, a large proportion of genes in mollusk species do not share orthologous relationships, but rather represent novel gene families [26]. Third, the high level of amino acid divergence to other, better studied invertebrate lineages and evolutionary distance to other organisms reduces the probability and quality of BLAST hits [26,27]. These points highlight the need for more genomic data from molluscs to increase our knowledge and facilitate genomic studies in this phylum.

## Conclusions

Here we have demonstrated the first transcriptome assembly of a non-model organism using *de novo *assembly of Solexa/Illumina data. However, results strongly depend upon assembly method and the number of high quality contigs is low. As assembly quality increases, including Solexa/Illumina on the list of sequencing platforms suitable for non-model species will greatly increase the possibilities for future evolutionary studies by decreasing costs and increasing transcriptome coverage. The widely distributed assembler VELVET produced by far the highest overall number of contigs. Investigating them more closely, however, revealed a large fraction of short contigs, redundant contigs, and a lower number of high quality BLAST hits in comparison to the other two assemblers. Even though contig sequence quality itself revealed to be good, as shown by the direct comparison to *R. balthica *mt genes, short contigs lead to fragmented coverage. OASES contigs closely match the BLAST results of NGEN. Thus it appears to be an almost equal alternative, and importantly, freely available. According to our findings the SeqMan NGEN software package performs best for short read assembly. For a cutoff value of <*e*^-10 ^and/or contig length > 200bp the long contigs resulted in an overall highest number of gene hits in different BLAST searches. The direct comparison to the mt genes showed that NGEN contigs almost covered the same amount of bp as Velvet, but due to long reads resulted in continuous coverage. However, long contigs also mean the loss of several short informative sequences (no contig hits for several mt genes) and contigs should be screened for unspecific sequence extensions. These drawbacks can be overcome by a meta-assembly of the first-order contigs sets. According to our results meta-assembly leads to longer contigs, higher number of hits in BLAST searches and should be considered as an additional step in contig assembly.

## Methods

### Sampling of snails

*Radix balthica *were caught from a highly inbred population (*F*_*IS *_= 0.53) from a small lake in Bavaria, Germany and kept for several generations in the laboratory. Snails were put in 500 ml jars under varying conditions (Additional file 5). Different aeration, life stages, temperatures, feeding conditions, salinity and temperature shock treatments were employed to cover a wide range of temperature related transcription patterns useful for later temperature involved studies. Two individuals per treatment were selected and pooled for cDNA synthesis (n = 24).

### cDNA library construction

RNA was isolated following the RNeasy maxi kit protocol (Qiagen) (Additional file 6 Figure S1). cDNA production and normalization was performed by GenXPro GmbH (Frankfurt, Germany). In order to optimize the recovery of full-length transcripts, Calf Intestinal Phosphatase (CIP, Ambion) was used to remove the 5'-phosphate from all RNA species except those with cap structure. Tobacco Acid Phosphatase (TAP, Ambion) was used to remove the cap structure, leaving RNA molecules with a 5' phosphate. An RNA oligonulceotide ("SceI-site") was then ligated to the afore capped RNAs. First strand synthesis was performed using SuperScript^® ^cDNA Synthesis System as described by the manufacturers, with SuperScript III as reverse transcriptase (Invitrogen) and a biotinylated oligodT with a T7 recognition site at the 5' end. The first strand product was used as template for a 20 cycles PCR reaction using primers specific for the T7 and the SceI-site. The PCR product was then normalized using the kamtchatka Crab Double Strand Specific Nuclease (DSN; Evrogen), as described by Zhulidov et al.[28] (Additional file 6 Figure S2). The normalized product was again amplified by PCR with 15 cycles and used as template for T7 -RNA polymerase amplification (Fermentas) for 5 hours. In order to select for longer transcripts, RNA > 600 bps was gel-selected and purified from the agarose gel (Additional file 6 Figure S3). The recovered RNA was fragmented by Zinc treatment. Illumina GA-II sequencing adapters were ligated to the fragments, as described by Illumina's Paired-End Sample Preparation Guide (catalogue number PE-930-1001). A 300-400 bp smear containing the cDNA fragments flanked by Illumina PE adapters was cut from an agarose gel and cDNA purified. Sequencing was performed on an Illumina GA-II using the Chrysalis 36 cycles v 3.0 sequencing kit, with one lane of 2 × 101 bp reads from both ends of the fragments ("paired ends") with an insert length of > 180 bp. The PhiX control lane revealed error rates between 3.16% for Read 1 and 4.07% for Read 2. Data basecall was performed using the Solexa GAPipeline 1.4.0. We chose to use the paired-end sequencing approach as it bears some additional advantages compared to normal one way sequencing. For one, information on two sequence reads being located in close vicinity is obtained and their position to one another known, second, if long enough these reads might be joined to one single read, longer than the usual read length.

### EST analysis and bioinformatics

The raw sequence data was quality checked and trimmed if five consecutive bases were of quality score "B", these plus the subsequent bases at the 3' end of the low-quality-base reads were discarded. T7 and Illumina adaptors were removed using an inhouse script.

EST-contigs were assembled using three different assemblers: a) VELVET short read assembler, Version 0.7.55 [29] as commonly used short read assembler, with following parameters: *k*-mer hash length 31, coverage = 1, read category = shortPaired; b) OASES short read assembler, Version 0.1.11 has recently been released as extension for Velvet, optimized for EST assembly (available at http://www.ebi.ac.uk/~zerbino/oases/). It uses preliminary VELVET assemblies with reduced setting options as input. Two additional VELVET assemblies were created with *k*-mer sizes 21 and 31, with read category set to shortPaired of the raw read data. The same *k*-mer sizes were additionally applied to the previously trimmed sequence set, which in both cases resulted in less contigs of smaller size (data not shown), and were thus excluded from further analysis.; c) SeqMan NGEN program (hereafter called NGEN) from Lasergene DNAStar software package (Madison, WI; http://www.dnastar.com/t-products-seqman-ngen.aspx) *k*-mer size = 31, Illumina reads and PairedEnd option turned on for the first contig assembly, with the Illumina raw reads. If not otherwise stated default parameters were used. Assemblies were run on computers with 32 Gb RAM, and finished within 24h.

As contigs created from the different assemblers differed in their read length, number of BLAST hits and position along a transcript, we additionally investigated whether a meta-assembly, thus the combined assembly of all four contig sets might increase contig information. The approach of combined assembly of several contig sets has already proven useful for 454-contigs obtained by different assemblers [30]. As our first-order contig analyses revealed adaptor remains on some contigs, all first-order contig sets were BLASTed against the T7 and Illumina adaptors to ensure high quality meta-assembly. If similarities were detected the according bases plus subsequent bases to the nearest sequence end were trimmed (this affected 3-7% for the Oases and Velvet as well as 35% of the NGen contigs). We chose to use NGEN for this task as it gave good results in our study but especially also for long read assembly [30]. Default parameters were used for the "other reads" option with *k*-mer size = 25.

Contigs from all assemblies can be found in Additional files 7, 8, 9, 10 and 11.

The BLAST program [31] was used to perform BLASTN and BLASTX homology searches in the following databases: RefSeq Invertebrates (Release38_11-11-2009) and UniProt protein database (UniProtKB/Swiss-Prot Release 2009_09) respectively. As the *Radix *genome has not been sequenced yet, we chose to use three indirect quality measures for an overall comparison of all contig sets. In addition we were able to inspect contig quality directly by making use of the recently obtained mitochondrial genome sequence of *R. balthica *[24]. The four different quality measures (three indirect and one direct) were defined and applied as follows:

a) Mapping accuracy has been shown to increase significantly with contig size (100bp vs. 300bp; [14]). We therefore compare the number of BLAST hits using a moderate cutoff value of < e^-5 ^to a smaller cutoff value of < e^-10 ^and/or contigs larger than 200bp.

b) The number of BLASTX hits against the EST database of *Biomphalaria glabrata *(NCBI EST databases, state march 2010), the only other gastropod available for comparison at the time with a large EST dataset. *B. glabrata *belongs to a sister-superfamiliy, which diverged about 180 million years ago. The EST data set with 86,121 ESTs of which 26,730 resulted in a hit with a cutoff value of < e^-5 ^and 5753 UniGens.

c) After removing redundant gene hits from each UniProt BLASTX data sets, only single hits with < e^-10 ^and larger 200bp were used to determine the number of conforming hits between contig sets produced by the three different assemblers. The higher the number of overlaps the better, follows the assumption that genes identified from contigs of two different assemblers should be trustworthy.

d) All contigs were subjected a BLASTN against the 13 mitochondrial genes of *R. balthica *(NCBI Accession No.: HQ330989). The overall number of hits, the number and percentage of identical bp between contigs and genes were determined and compared. Non-matching contig sections were visually scored as to their position at either the ends or middle of the contig. Additionally non-matching contig sections were separately aligned against the Illumina sequencing adaptor sequence to test whether they represent adaptor remains.

After evaluating the results of the above mentioned methods, we came to the conclusion that the best quality contigs obtained are the ones assembled by NGEN. We therefore produced an overview graph of contig average coverage versus contig length, and the gene ontology annotation (see below) for this contig set only. Contig average coverage (ac) was calculated by summing up the lengths of all sequences (ssl) building up one contig, divided by the contig length (cl), thus ac = ssl/cl.

### Gene ontology annotation

Assignment of gene ontology (GO) terms to the 2405 genes identified by both the NGEN and OASES*k*-mer 31 assembled contigs > 200bp was performed by importing the GO-numbers obtained by the above mentioned BlastX search against the UniProt database into BLAST2GO (version 2.4.4; http://www.blast2go.org/). BLAST2GO is an automated tool for the assignment of gene ontology terms and was designed for use with novel sequence data [32]. Categorization of the BLAST matches and construction of pie charts was conducted using the standard graph configurations including level two GO-terms only.

### SNP and sequencing error estimation

Reads of VELVET contigs that resulted in high quality hits against the mitochondrial genome of *R. balthica *were assembled against the latter using Geneious Pro 5.4.2 (http://www.geneious.com). SNP calling parameters were set to minimum coverage = 5, and minimum variant frequency = 0.2. Sites with at least 10x coverage and the presence of one single deviating nucleotide were scored as sequencing error.

### Repeat motifs

Microsatellite repeat motifs were identified using the program SPUTNIK (http://espressosoftware.com/sputnik/; [22]). SPUTNIK is a program based on C language which searches DNA sequence files for microsatellite repeats using a recursive algorithm. We then extracted di-, tri-, tetra-, and penta-nucleotide repeats with a minimum repeat length of six, respectively.

## Authors' contributions

BF organized and planned the research conducted the meta-assembly and other parts of data analysis and drafted the manuscript. CW conducted the NGEN and OASES assemblies, contributed to project planning and manuscript preparation. NK was involved in data analysis and conducted the VELVET assembly and BLAST searches. BR guided the sequencing and data analysis. MP designed the research project and contributed to data analysis and manuscript preparation. All authors have read and approved the final manuscript.

## Supplementary Material

Additional file 1 **Net coverage bp of *R. balthica *mitochondrial genes**. Coverage bp of *R. balthica *mitochondrial genes by contigs > 80% bp identity. Numbers in parentheses indicate the number of contigs with identities > 80% matching the respective gene, and thus are included in the net coverage calculation. Combined coverage: overall coverage, thus bp identity of all first assembly contigs taken together. Click here for file

Additional file 2 **Gene ontology distribution of *R. balthica *contigs**. Pie charts showing the distribution of contigs > 200bp, giving identical BLASTX gene hits for the NGEN and OASES*k*mer-31 assemblies, into the three main gene ontology categories. Click here for file

Additional file 3 **Microsatellite repeat motifs obtained from NGEN contigs**. List with NGEN contigs containing di-, tri-, tetra-, penta-repeat motives with a minimum repeat length of six, contig ID, repeat type, size, position and contig sequence. Click here for file

Additional file 4 **Screen shot contig assembly vs. mitochondrial genome**. The effect of a subsequent meta-assembly on contig redundancy is depicted here: a) Coverage of the mitochondrial gene COIII after the first separate assemblies and b) after the meta-assembly. Click here for file

Additional file 5 **Rearing conditions subjected to *Radix balthica *individuals**. Click here for file

Additional file 6 **RNA quality inspection**. RNA quality, RNA normalization, and RNA size selection gel pictures. Click here for file

Additional file 7 **VELVET assembly contigs**. Click here for file

Additional file 8 **OASES*k*mer-21 assembly contigs**. Click here for file

Additional file 9 **OASES*k*mer-31 assembly contigs**. Click here for file

Additional file 10 **NGEN assembly contigs**. Click here for file

Additional file 11 **Meta-assembly contigs**. Click here for file
