# Supplementary material for: Short read Illumina data for the de novo assembly of a non-model snail species transcriptome (Radix balthica, Basommatophora, Pulmonata), and a comparison of assembler performance
Source: BMC Genomics. 2011 Jun 16;12:317. doi: 10.1186/1471-2164-12-317 (PMC3128070; doi:10.1186/1471-2164-12-317)
Supplement: Additional file 1 — Net coverage bp of R. balthica mitochondrial genes. Coverage bp of R. balthica mitochondrial genes by contigs > 80% bp identity. Numbers in parentheses indicate the number of contigs with identities > 80% matching the respective gene, and thus are included in the net coverage calculation. Combined coverage: overall coverage, thus bp identity of all first assembly contigs taken together. [file 1471-2164-12-317-S1.DOC]

**Additional file 1:** Net coverage bp of *R. balthica* mitochondrial genes by contigs >80% bp identity. Numbers in parentheses indicate the number of contigs with identities >80% matching the respective gene, and thus are included in the net coverage calculation. Combined coverage: overall coverage, thus bp identity of all first assembly contigs taken together.

|  | original  gene | ORK21 | ORK31 | NGen | Velvet | combined  coverage | Meta |
| --- | --- | --- | --- | --- | --- | --- | --- |
| ND6 | 430 | 120 (1) | 0 | 0 | 274 (4) | 274 | 182 (1) |
| ND5 | 1498 | 256 (1) | 634 (3) | 753 (1) | 489 (7) | 763 | 750 (1) |
| ND1 | 912 | 613 (1) | 665 (1) | 633 (2) | 519 (6) | 843 | 714 (2) |
| ND4L | 357 | 136 (1) | 121 (1) | 0 | 92 (1) | 121 | 136 (1) |
| CYTB | 981 | 845 (1) | 806 (1) | 635 (3) | 622 (11) | 892 | 0 |
| COII | 677 | 155 (1) | 402 (2) | 0 | 560 (21) | 638 | 0 |
| ATP6 | 638 | 466 (2) | 408 (2) | 197 (1) | 162 (4) | 570 | 370 (2) |
| ND3 | 250 | 208 (1) | 0 | 0 | 94 (1) | 208 | 342 (1) |
| ND4 | 1328 | 1266 (3) | 1248 (3) | 1053 (5) | 1063 (23) | 1325 | 1389 (4) |
| ATP8 | 160 | 0 | 0 | 0 | 63 (1) | 63 | 0 |
| COIII | 788 | 0 | 0 | 788 (2) | 739 (96) | 788 | 788 (2) |
| ND2 | 874 | 257 (1) | 195 (1) | 141 (1) | 209 (2) | 266 | 262 (1) |
| COI | 1533 | 1010 (2) | 1048 (3) | 1410 (6) | 810 (37) | 1437 | 1200 (2) |
| SUM | 10,426 | 5332 | 5527 | 5610 | 5696 | 8184 | 6143 |
