# Supplementary figures and images for: Short read Illumina data for the de novo assembly of a non-model snail species transcriptome (Radix balthica, Basommatophora, Pulmonata), and a comparison of assembler performance
Source: BMC Genomics. 2011 Jun 16;12:317. doi: 10.1186/1471-2164-12-317 (PMC3128070; doi:10.1186/1471-2164-12-317)

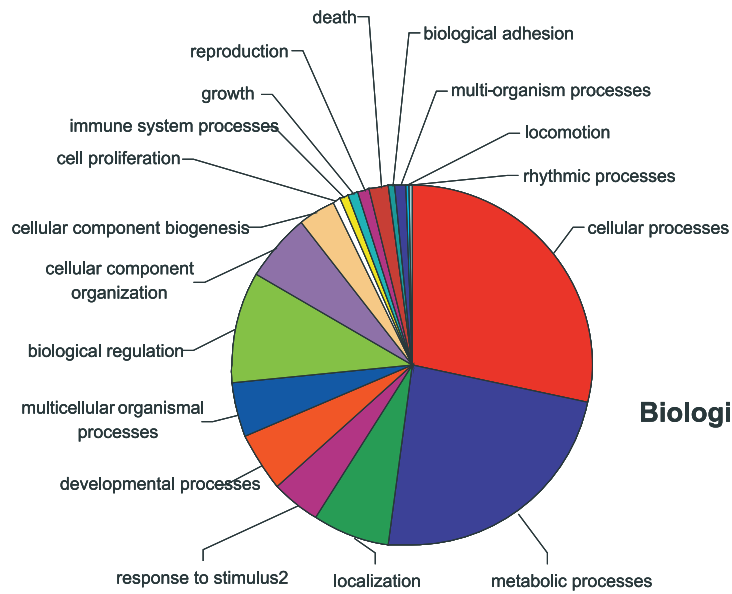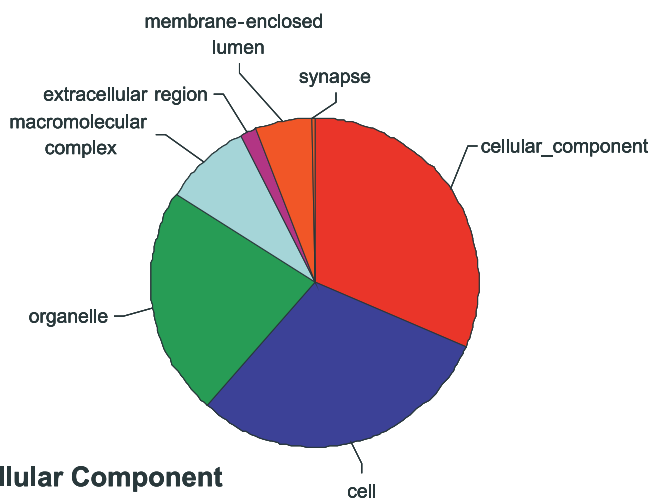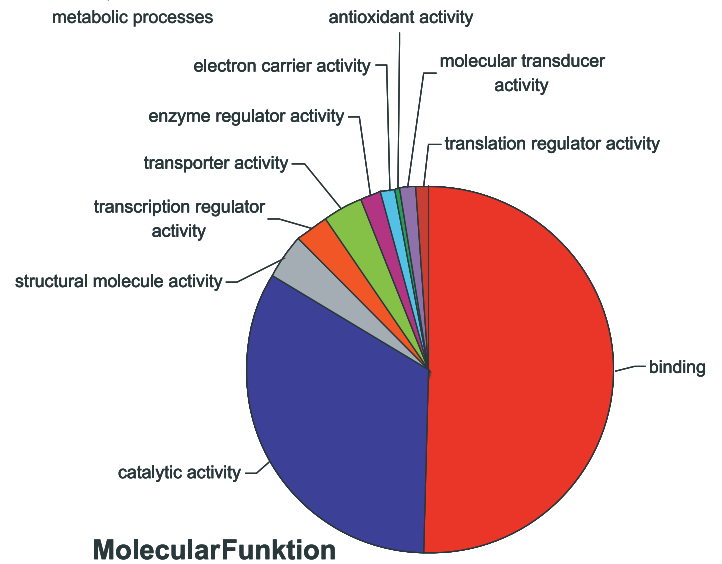

Supplement: Additional file 2 — Gene ontology distribution of R. balthica contigs. Pie charts showing the distribution of contigs > 200bp, giving identical BLASTX gene hits for the NGEN and OASESkmer-31 assemblies, into the three main gene ontology categories. [file 1471-2164-12-317-S2.PDF]

Mt genome coverage COIII after a) first- and b) meta-assembly.

a)

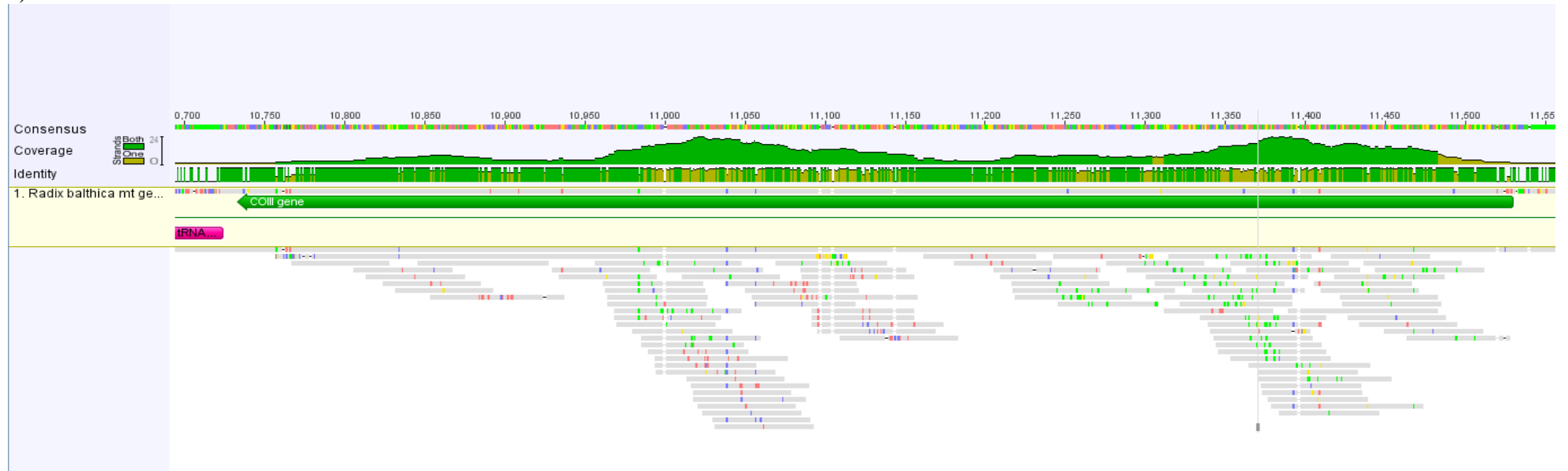

b)

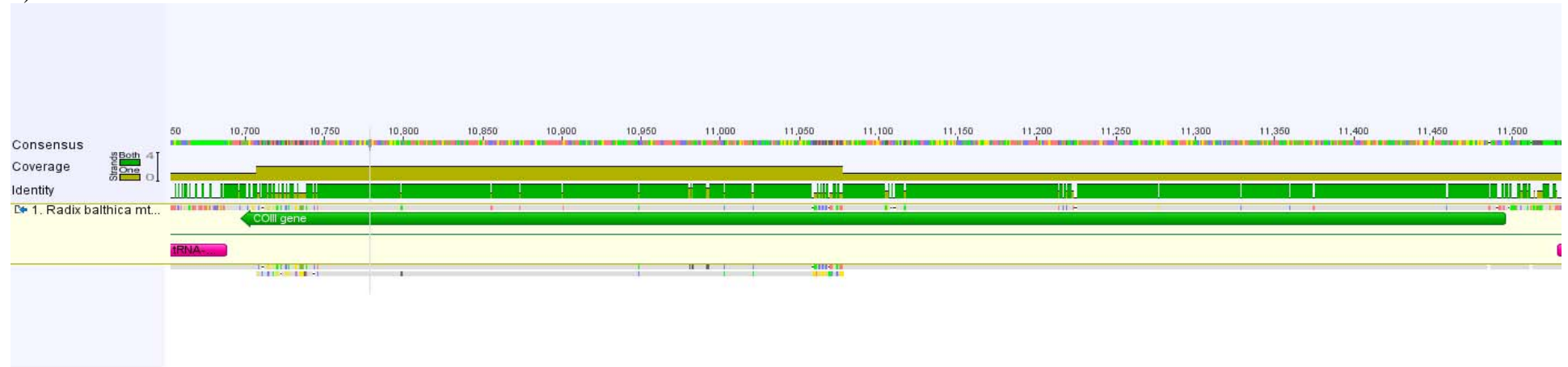

Supplement: Additional file 4 — Screen shot contig assembly vs. mitochondrial genome. The effect of a subsequent meta-assembly on contig redundancy is depicted here: a) Coverage of the mitochondrial gene COIII after the first separate assemblies and b) after the meta-assembly. [file 1471-2164-12-317-S4.PDF]
