# Supplementary material for: Short read Illumina data for the de novo assembly of a non-model snail species transcriptome (Radix balthica, Basommatophora, Pulmonata), and a comparison of assembler performance
Source: BMC Genomics. 2011 Jun 16;12:317. doi: 10.1186/1471-2164-12-317 (PMC3128070; doi:10.1186/1471-2164-12-317)
Supplement: Additional file 5 — Rearing conditions subjected to Radix balthica individuals. [file 1471-2164-12-317-S5.DOC]

**Additional file** 5: Rearing conditions subjected to *Radix balthica* individuals that were used for RNA extraction. Light conditions 16L:8D.

| Age | °C | Food | Aeration | Else | Comment |
| --- | --- | --- | --- | --- | --- |
| Young adult | 20 | Ad lib | Yes | - | Usual rearing conditions |
| Young adult | 20 | Ad lib | No | - | Anoxic conditions |
| Young adult | 20 | Min | Yes | - | Starving conditions |
| Young adult | 20 | Ad lib | Yes | Increased salinity (2%) | Brackish water |
| Young adult | 20 | Ad lib | Yes | Temp shock 20min at 30°C |  |
| Young adult | 20 | Ad lib | Yes | Temp shock 20min at 5°C |  |
| Young adult | 12 | Ad lib | Yes |  | Cold |
| Young adult | 12 | Min | Yes |  | Cold/starving |
| Young adult | 26 | Ad lib | yes |  | Warm |
| Young adult | 26 | Ad lib | no |  | Warm/anoxic |
| Juvenile | 20 | Ad lib | Yes |  | Developing |
| Old adult | 20 | Ad lib | Yes |  | Mature, likely fertilized |
