# Supplementary material for: Short read Illumina data for the de novo assembly of a non-model snail species transcriptome (Radix balthica, Basommatophora, Pulmonata), and a comparison of assembler performance
Source: BMC Genomics. 2011 Jun 16;12:317. doi: 10.1186/1471-2164-12-317 (PMC3128070; doi:10.1186/1471-2164-12-317)
Supplement: Additional file 6 — RNA quality inspection. RNA quality, RNA normalization, and RNA size selection gel pictures. [file 1471-2164-12-317-S6.DOC]

Figure S1: Caliper picture RNA quality check

Figure S2: Gel picture of amplified cDNA a) prior to and b) after normalization

a)

Lane 1 Lane 2 Lane 3


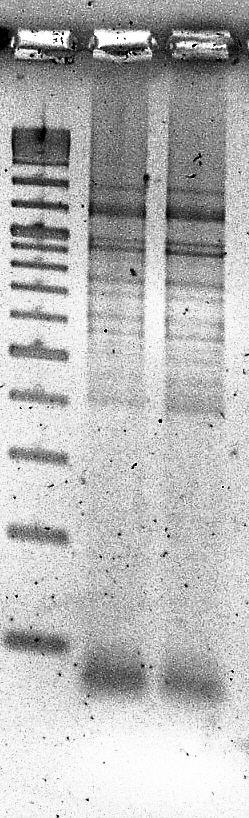


b)

Lane 1 Lane 2 Lane 3 Lane 4 Lane 5 Lane 6


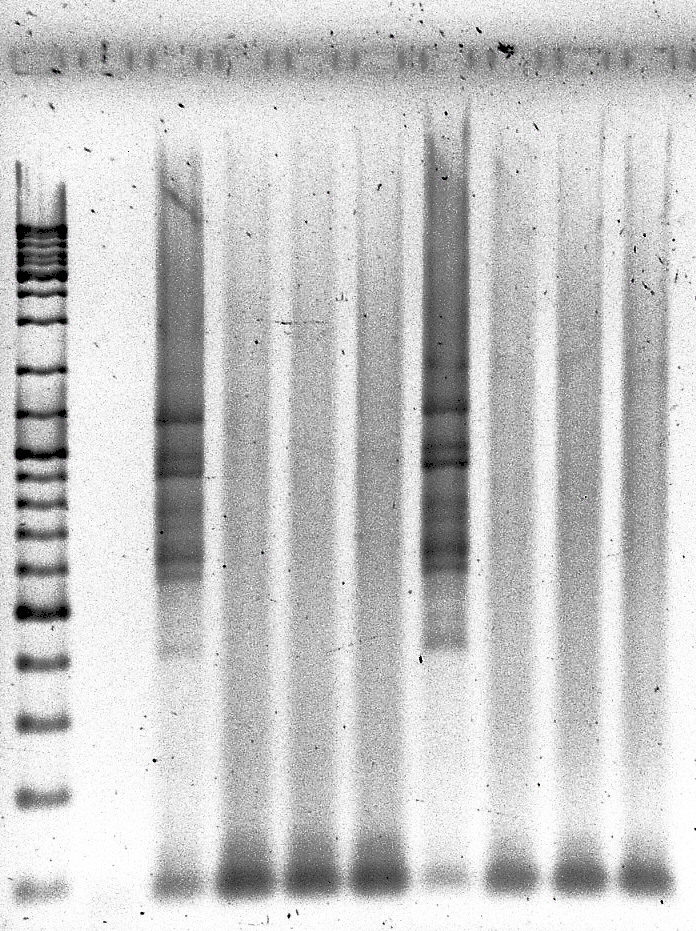


Lane 1: DNA Ladder mix Gene Ruler Fermentas 0.5 ug/ul

Lane 2: empty

Lane 3: no DSN

Lane 4: DSN treatment (undiluted Enzyme)

Lane 5: 1:2 diluted DSN treatment

Lane 6: 1:4 diluted DSN treatment

Figure S3: Size-selection


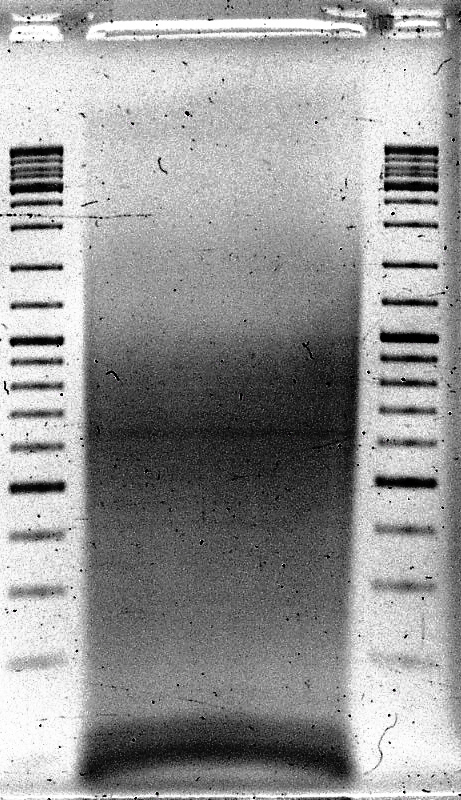


mRNA above 600 bp was cut from gel, followed by RNA-seq protocol.
